# Atipamezole Reverses Cardiovascular Changes Induced by High-Dose Medetomidine in Cats Undergoing Sedation for Semen Collection

**DOI:** 10.3390/ani13121909

**Published:** 2023-06-07

**Authors:** Anna-Lea R. Diggelmann, Marco Baron Toaldo, Rima N. Bektas, Etienne Furthner, Iris M. Reichler, Annette P. N. Kutter

**Affiliations:** 1 Section of Anaesthesiology, Department for Clinical Diagnostics and Services, Vetsuisse Faculty, University of Zurich, 8057 Zurich, Switzerland; 2 Clinic for Small Animal Internal Medicine, Division of Cardiology, Vetsuisse Faculty, University of Zurich, 8057 Zurich, Switzerland; 3 Clinic of Reproductive Medicine, Department for Farm Animals, Vetsuisse Faculty, University of Zurich, 8057 Zurich, Switzerland

**Keywords:** echocardiography, cardiac troponin I, alpha2 adrenoreceptor agonist, haemodynamics, semen collection

## Abstract

**Simple Summary:**

Medetomidine is a sedative agent widely used in veterinary medicine. It acts on alpha2 adrenoreceptors and thus also affects the cardiovascular system. Most frequently observed changes include a reduction in the heart rate and an increase in arterial blood pressure. In higher dosages, medetomidine can be used as sole agent for semen collection via urethral catheterisation in cats. Due to the marked haemodynamic effects, the potential risk of the repeated administration of high-dose medetomidine in breeding cats needs to be considered. Sedation with medetomidine can be reversed through its antagonist atipamezole. In this study, echocardiography was used to assess the cardiovascular changes after medetomidine administration. Different parameters were measured during sedation and after reversal with atipamezole. To further investigate the potential negative effects of repeated sedation on the heart muscle, the concentrations of a specific cardiac biomarker were determined. Medetomidine administration markedly altered cardiovascular function and led to a reduction in the heart rate and heart performance. However, reversal with atipamezole resulted in a normalization of haemodynamics. Repeated sedation did not lead to any adverse effects and no sustained damage of the heart muscle by means of elevated cardiac biomarker concentrations could be detected.

**Abstract:**

This study aimed at describing the change in echocardiographic variables after high-dose medetomidine and the reversal with atipamezole in six cats undergoing sedation for semen collection. Further cardiac Troponin I (cTnI) concentration and the effect of repeated sedation were assessed. Echocardiography was performed before and 20 min after sedation with 0.1 mg/kg medetomidine intramuscularly (IM) for urethral catheterisation. Prior to epididymectomy, S-ketamine was administered intravenously. Twenty minutes after reversal with 0.5 mg/kg atipamezole IM, the third echocardiography was performed. Sedation with medetomidine and reversal with atipamezole was repeated on day 7, 14, 21 and 28. Heart rate (HR) and rhythm were monitored throughout all sedations. On day 0 and 28 cTnI concentrations were measured before and after the procedure. After normality testing, the values were compared over time. The administration of medetomidine led to a marked reduction in HR, cardiac output and ventricular systolic function and a significant increase in left ventricular dimensions. Rhythm abnormalities, such as ventricular premature complexes and idioventricular rhythm, could be observed. The administration of atipamezole completely reversed sedation and the changes in haemodynamic variables. No significant increase in cTnI concentrations could be detected, although two out of six cats showed values above the reference range.

## 1. Introduction

Medetomidine is an alpha2 adrenoreceptor agonist widely used in veterinary medicine as a sedative, analgesic and muscle relaxing agent [1]. Because medetomidine is a lipophilic compound, it is well absorbed after intramuscular (IM) administration [2], which allows for rapid and effective sedation in cats [3].

Medetomidine is also reported as sole sedative agent for semen collection in cats. A postsynaptic alpha2-mediated contraction of the deferent duct leads to sperm release into the urethra, which then can be collected via urethral catheterisation [4]. Higher dosages (0.1–0.14 mg/kg) than normally used in clinics are needed to assure good semen quality in terms of volume, concentration, the total number of spermatozoa and the quality of the movement [5]. As the protocol is repeatedly used in breeding cats, the potential risks of repeated administration of high-dose medetomidine need to be considered.

Medetomidine has marked haemodynamic effects. The stimulation of postsynaptic alpha2 adrenoreceptors causes vasoconstriction with a subsequent increase in systemic vascular resistance (SVR) [6]. Both the baroreceptor mediated reflex response to the vasoconstriction and the centrally mediated sympatholysis lead to bradycardia [1]. In clinically normal cats, the decrease in heart rate (HR) and the increase in SVR have been shown to result in a marked reduction in cardiac output [7,8]. A former study examined the effects of high-dose medetomidine (0.13 mg/kg IM) on the feline heart using echocardiography and found significant and clinically relevant changes [9]. In cats, the alpha2 adrenoreceptor antagonist atipamezole has been shown to reverse sedation and HR changes caused by medetomidine (0.08 mg/kg IM) [10].

Cardiac Troponin I (cTnI) is considered the most specific biomarker for myocardial damage in humans [11], cats and dogs [12]. cTnI is a cardiac specific protein found as part of the troponin–tropomyosin complex in striated muscle cells and regulates the muscle contraction by inhibiting the interaction of actin and myosin in the absence of Ca^2+^ [13]. Normally, cTnI is found only in low concentrations in the bloodstream of healthy cats [14], the normal range is reported to be 0–0.09 ng/mL [15]. This biomarker is released shortly after damage and concentrations correlate with the degree of myocardial injury [16]. cTnI leaks into the blood stream when sarcolemma permeability increases and the contractile apparatus of the myocardial cell is disrupted [17] and can be measured both with enzyme-linked immunoabsorbant assays (ELISA) [18] or with a point of care device from human medicine, as the protein is highly conserved among species [19]. However, cTnI cannot discern different underlying diseases [20].

This study aimed at describing the change in echocardiographic variables after high-dose medetomidine and the reversal with atipamezole in six cats undergoing sedation for semen collection. Further cardiac Troponin I (cTnI) concentration and the effect of repeated sedation were assessed. The hypothesis was that changes in echocardiographic variables would be found after the administration of a high dose of medetomidine and that the changes would not be completely reversed after the administration of atipamezole. We also hypothesized that sedation and reversal would increase cTnl levels. The second hypothesis was that the repetitive administration of high-dose medetomidine would cause prolonged sedation and more profound cardiovascular effects over time.

## 2. Materials and Methods

### 2.1. Animals

Six intact, purpose-bred male cats of 5.5 (2–8) (median (range)) years old and weighing 5.58 (4.33–6.28) kg were enrolled in the current study. All animals underwent physical examination to rule out major health problems and were considered healthy. Food was withheld for 12 h prior to sedation with free access to water.

The study was approved by the Veterinary Office of the Canton of Zurich, Switzerland (license number ZH093/2020), and followed the guidelines of the Swiss Animals Protection Law and was a prospective experimental trial.

### 2.2. First Part of Study (Semen Collection, Epididymectomy and Echocardiography)

On day 0, each cat was administered 0.1 mg/kg medetomidine (Medetor^®^, Virbac, Glattbrugg, Switzerland) IM into the infraspinatus muscle. Cats were placed back in a carrier and monitored for depth of sedation. As soon as lateral recumbency was achieved, an intravenous catheter was placed for the purpose of blood collection, drug and fluid administration. All cats received Ringer Acetate (Ringer Acetat, Bichsel AG, Unterseen, Switzerland) at 5 mL/kg/h.

For semen collection for a concurrent study assessing the effects of epididymectomy [21], an urethral catheter was placed with the cats in lateral recumbency. Thereafter, to reach adequate level of anaesthesia and prevent the perception of the surgical stimulus during epididymectomy, S-ketamine (Ketaset^®^, Zoetis, Delemont, Switzerland) (2.25 (1–3) mg/kg) was administered intravenously (IV) and cats were moved to dorsal recumbency. Subsequently to the successful completion of the surgical procedure, all cats received meloxicam 0.2 mg/kg (Metacam^®^, Boehringer Ingelheim, Basel, Switzerland) subcutaneously for postoperative analgesia.

Twenty minutes at the earliest after S-ketamine administration, the cats were antagonised with atipamezole 0.5 mg/kg (Revertor^®^ Virbac, Glattbrugg, Switzerland) IM into the infraspinatus muscle. The time between all injections was recorded. The times needed to retrieve sternal recumbency and standing position were not consistently recorded. The attending board-certified anaesthesiologist (AK, RB) recorded whether the reversal of sedation was complete after all sedations.

The data collection of the heart rate (HR), heart rhythm and echocardiographic examinations was performed before sedation (baseline, BL), 20 min after medetomidine administration (MED) and 20 min after reversal with atipamezole (ATI), respectively.

During the echocardiography, a continuous electrocardiographic (ECG) recording was obtained using a standard lead II tracing. Throughout the whole procedure, HR and heart rhythm using a standard lead II tracing, the colour of mucous membranes and the saturation of haemoglobin with oxygen (SpO_2_) were monitored continuously on a multiparameter monitor (Cardiocap/5, GE, Datex-Ohmeda, Anandic, Feuerthalen, Switzerland) and recorded every 5 min when available. If SpO_2_ measurements were lower than 95% or not measurable, the cats received 2 L/minute of oxygen flow by with a face mask.

The cardiac rhythm was visually assessed from the ECG by the attending anaesthesiologist based on the following criteria: normal sinus rhythm; sinus arrhythmia—visible variation of the R-R interval; ventricular premature complex (VPC)—premature wide and bizarre QRS complex without associated P-wave and HR > 100 beats/minute; and idioventricular rhythm—only wide and bizarre QRS complexes without associated P-wave and HR < 160 beats/minute.

Echocardiographic examinations were carried out by one single board-certified cardiologist (MBT). Cats were examined in right and left lateral recumbency following a standard protocol and using an ultrasound machine (CX50, Philips Health Care, Horgen, Switzerland) equipped with a multifrequency (S12-4) phased-array transducer. Two-dimensional, M-Mode, colour, pulsed wave and continuous wave Doppler as well as tissue Doppler images and videoclips were obtained using methods described elsewhere by one of the authors [9,22,23]. Echocardiographic studies were anonymized and transferred to an external hard disk for subsequent analysis using a dedicated workstation (TOMTEC ARENA, TOMTEC, Unterschleissheim, Germany). All echocardiographies were analysed in a randomized order at the end of the study, by the same cardiologist, who was blinded to the cats’ identity. Each parameter represents the mean of measurements of three consecutive cardiac cycles.

Baseline cTnI concentrations were measured in blood withdrawn from a venous catheter placed as soon as possible after sedation with medetomidine (D0_T0). Blood was sampled into a 1 mL syringe and used directly to fill the cartridges (iStat cTnI cartridge, Abott Medical AG, Zurich, Switzerland) for the i-STAT® device (Abott iStat Portable Clinical Analyzer, Abott Medical AG, Zurich, Switzerland). This point of care two-site enzyme-linked immunosorbent assay has an analytical range of 0–50 ng/mL. cTnI concentrations below 0.09 ng/mL were considered normal. The measurement of cTnI was repeated after the completion of the study before the catheter was removed (D0_T1).

### 2.3. Second Part of the Study (Semen Collection)

Sedation with 0.1 mg/kg medetomidine IM, its reversal with 0.5 mg/kg atipamezole IM and urethral catheterisation for semen collection was repeated at the interval of seven days (day 7, 14, 21, 28). Data (HR and heart rhythm) were collected before sedation (baseline, BL), 20 min after the administration of medetomidine (MED) and 20 min after reversal with atipamezole (ATI). The HR was retrieved by listening to the heart with a stethoscope and heart rhythm was assessed as described above. Measurements of cTnI concentrations were repeated on day 28 at the same two timepoints as on day 0 (T0_D28, T1_D28).

### 2.4. Statistical Analysis

All measured and calculated data were transmitted into an Excel Sheet (Microsoft Office 2019, Microsoft Corporation, Redmond, WA, USA). Data were analysed using GraphPadPrism 9.4.1 (GraphPad Software Inc., San Diego, CA, USA). Statistical tests between groups were only performed when values for ≥5 cats were available and for selected variables to avoid the multiple testing of dependent variables. The normal distribution of the variables was assessed using the Shapiro–Wilk test and the inspection of the normality plot. If the variables were normally distributed, a repeated measurement one-way ANOVA was used to compare the cardiovascular variables at BL, MED and ATI. For the comparison of non-normally distributed values, Friedman’s test was used. If differences were considered significant, Dunnett’s test or Dunn’s test (as appropriate) was used for the comparison of MED and ATI to BL. The cTnI values retrieved at the different time points were compared with the values on day 28 with a two-way ANOVA. A *p*-value < 0.05 was considered significant.

## 3. Results

On day 0, all six cats recovered uneventfully from the procedure and neither persistent sedation nor changes in haemodynamic function could be observed at recovery. One cat had a heart murmur on auscultation, which was not associated with any structural cardiac abnormality at BL echocardiography.

In all cats, visible sedation and lateral recumbency were very rapidly achieved and SpO_2_ was not measurable throughout the whole duration of sedation. All cats received 2 L/minute of oxygen flow by with a face mask. Semen collection and epididymectomy were performed successfully in all cats and the results of this part of the study are presented elsewhere [21].

The second echocardiography exam MED started 21 (19–24) min after the administration of medetomidine IM. Thereafter, cats received 2.25 (1–3) mg/kg S-ketamine IV prior to the epididymectomy. Atipamezole was administered IM 62 (49–81) min after medetomidine and 20 (20–35) min after S-ketamine and resulted in the rapid and full reversal of sedation, the return of pink mucous membranes and no signs of excitation in all cats. The third echocardiography ATI started 22 (15–31) min after atipamezole and 43 (35–51) min after S-ketamine administration.

The results of the statistical analysis of the heart rate and selected echocardiographic variables are presented for BL, MED and ATI in Table 1. In Table 2, all echocardiographic variables are presented.

At MED, heart rate and parameters representing systolic function were significantly reduced and systolic left ventricular dimensions significantly increased. Diastolic left ventricular dimensions and parameters describing diastolic function were also significantly different to BL. At ATI, no significant differences to BL were found.

In three out of six cats, an aortic insufficiency was observed at MED, and in one cat, a mitral valve insufficiency was observed at MED. All insufficiencies in all cats disappeared after atipamezole administration.

**Figure 1:**
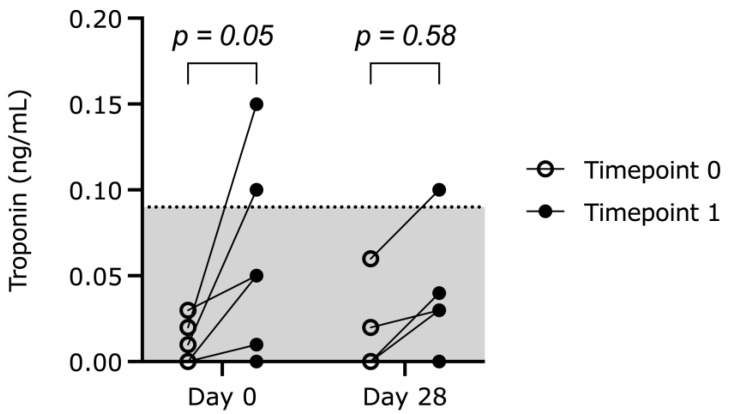
Individual cTnI concentrations on day 0 (D0) and day 28 (D28) of six healthy male cats undergoing repeated sedation on day 0, 7, 14, 21 and 28. Measurements were performed directly after intramuscular administration of 0.1 mg/kg medetomidine (timepoint 0, T0) and at least 20 min after intramuscular administration of 0.5 mg/kg atipamezole (timepoint 1, T1). The reference range is underlaid in grey (0–0.09 ng/mL).

For the cTnI concentrations, neither treatment (Figure 1) nor day (*p* = 0.26) had a significant effect. All cats had a cTnI concentration within reference range at D0_T0 and D0_T28. However, two out of six cats had cTnI concentrations above the reference range at T1 on day 0 and one of these cats also at T1 on day 28.

The HR at BL, MED and ATI and the occurrence and type of arrhythmia on day 0–28 are reported in Table 3 and Table 4, respectively. On day 0, the assessment of cardiac rhythm revealed VPC and an idioventricular rhythm after the administration of medetomidine in three out of six cats (Table 4). On the following four study days, similar patterns of arrhythmia could consistently be observed in the single individuals. All cats showed arrhythmias at least once after medetomidine administration.

All six cats recovered uneventfully from the following four sedations, where atipamezole was given 24 (17–37) min after medetomidine.

## 4. Discussion

During sedation with a high dose of medetomidine, the observed significant changes in cardiac volumes and systolic and diastolic function can be considered severe and clinically relevant. However, all values returned to baseline after the administration of atipamezole and all six cats recovered uneventfully from all five deep sedations. The individually increased cTnI values after medetomidine and atipamezole administration might represent a sign for acute myocardial damage. However, the changes did not reach significance and baseline concentrations measured on day 28 were back within reference range.

The administration of 0.1 mg/kg medetomidine IM resulted in significant and marked bradycardia as well as a reduction in stroke volume and cardiac output. A similar reduction in the described parameters has already been observed for both lower (0.01–0.02 mg/kg IM) [7,8] and higher dosages of medetomidine (0.13 mg/kg IM) and dexmedetomidine (0.04 mg/kg IM) in cats [9,24].

The observed increase in end-diastolic and end-systolic left ventricular dimensions (Table 1) reflects the previously reported echocardiographic changes in pre- and afterload following high-dose (dex)medetomidine administration in cats [9,24]. Typically, bradycardia causes a higher preload, which could be described by an increase in end-diastolic volume (EDV) in the present study. However, this did not result in an elevated stroke volume. The observed decrease in stroke volume after medetomidine administration can be explained by the concurrent left ventricular systolic dysfunction (reduced ejection fraction, decreased longitudinal ventricular function (MAPSE) and decreased velocity of the myocardium during systole (MV s). The right ventricular fractional area change was significantly decreased after medetomidine, indicating concurrent systolic dysfunction of the right ventricle. In autonomically blocked dogs, medetomidine had no direct impact on myocardial contractility [25]. Thus, systolic dysfunction is most likely caused by either the indirect reduction in sympathetic tone or the observed increase in afterload, which might secondarily affect systolic function with preserved intrinsic contractility. Due to large changes in pre- and afterload and due to the non-invasive assessment method, no definitive conclusions about changes in intrinsic contractility can be made.

Medetomidine appears to also affect diastolic variables with a possibly decreased lusitropy. A significant increase in the isovolumic relaxation time and decrease in the left ventricular early inflow wave velocity (E wave) was found (Table 1). However, the measurements of diastolic variables are influenced by many different factors, especially the observed changes in heart rate, left ventricular systolic function and pre- and afterload [26]. Thus, it is not possible to determine with non-invasive measurements, such as echocardiography, if medetomidine has a direct or indirect effect on myocardial relaxation and compliance during diastole [26].

The parameters of left atrial systolic function appeared to be negatively affected by medetomidine administration, as a significant decrease in left atrial fractional shortening could be observed. However, we need to consider that the parameters of left atrial function are also influenced by left ventricular diastolic function and changes in loading conditions. Furthermore, the left ventricular late inflow wave velocity (A wave), which is another marker for the systolic function of the left atrium, could not be measured in all cats at BL and ATI, as due to high heart rates, the E and A wave were fused.

Of the six cats, three presented with aortic and one with mitral valve regurgitation after the administration of medetomidine. This was a lower number than observed in other studies (8/8 cats with aortic and 5/8 cats with mitral valve regurgitation after medetomidine 0.13 mg/kg IM [9] and 12/14 cats with aortic and 14/14 cats with mitral valve regurgitation after dexmedetomidine 0.04 mg/kg IM [24]). The increase in mitral and aortic valve insufficiencies was likely caused by both an increased afterload due to peripheral vasoconstriction and an increased preload with mitral valve annulus stretch. As neither systemic or pulmonary blood pressure nor right or left atrial pressure were measured during this study, we cannot define the casual mechanisms in more detail.

The administration of 0.5 mg/kg atipamezole IM resulted in rapid decline in clinical sedation and restoration of haemodynamic function in all six cats without causing any obvious adverse reactions. Atipamezole is known to markedly reduce recovery times after medetomidine sedation [27]. The administration of atipamezole (0.2 mg/kg IM) following sedation with medetomidine (0.08 mg/kg IM) in cats led to the complete reversion of sedation, HR, pulse character and mucous membrane colour compared to baseline with full antagonism completed within 15 min [10]. In the current study, all echocardiographic variables at timepoint ATI had returned back to baseline. Pre- and afterload was normalized and measurements for cardiac output and the stroke volume of the left ventricle returned to baseline, indicating the recovery of forward blood flow. Systolic and diastolic function improved towards baseline. Mitral and aortic valve regurgitation were abolished in all cats. These results are in contrast to another echocardiographic study, evaluating the effect of 0.04 mg/kg dexmedetomidine IM and its reversal with atipamezole 0.4 mg/kg IM in cats [24]. Although HR and systolic function measured 2h after atipamezole administration increased significantly compared to during dexmedetomidine sedation, these parameters remained significantly different from baseline, indicating only the partial reversal of the effects of dexmedetomidine [24]. It is possible that the different timing of the measurements after atipamezole in these two studies led to this discrepancy.

The direct visual assessment of cardiac rhythm on the ECG monitor revealed marked bradyarrhythmia with ventricular premature complexes and idioventricular rhythm after the administration of high-dose medetomidine (0.1 mg/kg IM). In another study that used dexmedetomidine (0.04 mg/kg IM) in cats, bradyarrhythmias and ECG abnormalities were classified as minor [24]. Studies comparing the outcomes of dexmedetomidine to medetomidine administration in cats [3,10] reported no differences concerning the effects on the cardiovascular system. In the current study, the occurrence and type of bradyarrhythmia varied among individual cats, but interestingly, individuals quite consistently showed the same changes in rhythm during the following sedations. The most obvious explanations for the occurrence of bradyarrhythmias are the depressed sympathetic tone and the reflex sinus bradycardia. It is not yet known whether arrhythmia could also be caused by myocardial hypoxaemia and subsequent myocardial damage following alpha2-mediated haemodynamic effects [28]. Owing to a reduction in cardiac output, systemic oxygen delivery decreases after medetomidine. This led to a higher oxygen extraction in the organism [29] and the heart [30] in dogs. The increased myocardial oxygen extraction indicates a reduced coronary blood flow attributable to an alpha2-mediated vasoconstriction of the coronary vessels [30]. In the current study, cTnI was used as a marker to assess possible myocardial damage caused by medetomidine, S-ketamine (day 0) and the reversal with atipamezole. The amount of cTnI released stands in relation to the extent of myocardial necrosis [16]. It is still unclear whether increased cTnI concentrations can solely be associated with transient and reversible myocardial damage or always go in hand with irreversible necrosis [11]. In contrast to our results, Cote et al. found a significant increase in cTnI concentrations 2 h after atipamezole administration (0.4 mg/kg IM) following dexmedetomidine (0.04 mg/kg IM) in cats [24]. The values in that study increased from 0.02 (0.014–0.029) ng/mL (mean + 95% CI) to 0.073 (0.036–0.146) ng/mL and were still significantly higher compared to baseline 24 h after dexmedetomidine. This supports a possible negative effect of alpha2 agonists on the myocardium, suggesting no benefit of dexmedetomidine over medetomidine.

Unmeasurable oxygen saturation during sedation is most likely attributable to the fact that the use of pulseoximetry is limited by the presence of severe vasoconstriction. The investigation of the respiratory effects of medetomidine revealed no significant change in PaO_2_ [29]. However, all cats were administered oxygen flow by over a mask as soon as oxygen saturation was no longer measurable.

Although each administration of high-dose medetomidine led to marked reduction in heart rate and bradyarrhythmia, all six cats recovered from the four following sedations on day 7–28 without obvious side effects. Atipamezole was effective in reversing clinical sedation and bradycardia after repeated sedation. It has been stated previously in dogs that the marked effect on the cardiovascular system seems to be dose-independent with doses ≥ 0.005 mg/kg medetomidine intravenously. Higher dosages led to longer duration of action but cardiovascular depression did not increase further [31]. The repeated administration of high-dose medetomidine did not result in clinically obvious changes in the decrease in HR and the occurrence and severity of arrhythmias. If transient myocardial hypoxaemia occurred during sedation, it probably did not lead to sustained myocardial damage. It is debatable whether the cTnI concentrations on day 28 that were within reference range signify that one week in between sedations was sufficient to recover from a sedation with high-dose medetomidine in these six young healthy cats. It was reported that increased cTnI values only decrease to normal 14 days after myocardial injury. Therefore, a significant later rise in cTnI values some days after the preceding sedation would theoretically still have been detectable on day 28. The assessment of the impairment of the cardiovascular system with subsequent or later consequences for the animal could not be evaluated; however, at day 50 of the concurrent study, no cat presented with abnormalities on clinical examination.

For the present study, some limitations need to be considered. First of all, only six cats were used, and the study could thus be underpowered. The small sample size was defined by the concurrent study [21]. Nevertheless, the clinically and statistically significant results at MED show that the number of animals was sufficient to demonstrate the effects of medetomidine and its reversal with atipamezole on the cardiovascular system. However, the rise in cTnI concentration did not reach significance at the four timepoints analysed in one analysis, although some cats showed a rise in this parameter and values above reference range (Figure 1). Another contributing factor for not reaching significant results for cTnI could be the early analysis of the second blood sample shortly after the administration of atipamezole. To increase the possibility of finding a rise in the cTnI concentration, the second blood sample was retrieved as late as possible. Studies from human medicine indicate an increase 2–3 h after the initial myocardial insult with peak concentrations being reached within 18–24 h [16]. Therefore, we cannot exclude that the cTnI concentration rose much higher after the study and that the increase went undetected. However, due to the narrow time plan determined by the concurrent study, no later measurements were possible. For the epididymectomy variable dosages of S-ketamine were used. Theoretically, the sympathomimetic action of S-ketamine could have influenced the measurements of the third echocardiography on day 0. In another study evaluating the recovery after intramuscular anaesthesia with 0.03 mg/kg medetomidine, 6 mg/kg S-ketamine and antagonization with 0.15 mg/kg atipamezole directly after neutering of male cats, all cats were standing 22 ± 6 min thereafter. The recovery was much faster compared to 10 mg/kg racemic ketamine [32]. We administered a relatively lower dose (1–3 mg/kg) of S-ketamine intravenously, both resulting in a further reduction in the duration of action compared to the previous study. Furthermore, there were at least 43 (35–51) min between the administration of S-ketamine and the third echocardiography. As no signs of remaining ketamine such as twitching, nystagmus or increased muscle tone were present at reversal, all S-ketamine had probably been redistributed within the time between administration and the third echocardiography. The effect of any remaining S-ketamine was likely of minor clinical importance. The low dose of ketamine led to a relatively superficial anaesthesia depth, not allowing for the intubation of the cats. Neutering with medetomidine-ketamine combinations without performing endotracheal intubation is common practice in cats. Although the airway is not protected, side effects of endotracheal intubation commonly reported in cats can be avoided [33]. A further limitation poses the analysis of the ECG recordings, as they were only visually assessed by the attending anaesthesiologist.

Based on the current study, the administration of the full dose of the reversal agent 17–81 min after 0.1 mg/kg medetomidine IM with or without concurrent S-ketamine reversed sedation and cardiovascular changes in cats and did not lead to any observed morbidities or mortalities. The current study supports the use of atipamezole to reverse deep sedation for semen collection in healthy young cats without pre-existing cardiovascular comorbidities. Therefore, the reversal of sedation is emphasized to reduce the duration of cardiovascular depression, especially when high dosages of medetomidine are used. However, we cannot conclude whether the significant cardiovascular effects of repeated sedation with high-dose medetomidine may be detrimental in non-healthy cats. It is known that subclinical cardiac and vascular disease in cats can long go undetected. Therefore, a previous clinical examination and cardiac ultrasound should be performed. Although medetomidine has been shown to be a suitable sedative agent in cats with outflow tract obstruction due to left ventricular hypertrophy [34], it would be ethically questionable to assess the reported dosages in cats with pre-existing myocardial disease for semen collection.

## 5. Conclusions

The intramuscular administration of 0.1 mg/kg medetomidine in cats resulted in a marked bradycardia, a reduction in cardiac output and systolic function and a significant increase in left ventricular dimensions. Rhythm abnormalities, such as ventricular premature complexes and idioventricular rhythm, could be observed. The intramuscular administration of 0.5 mg/kg atipamezole IM completely reversed sedation and led to a restoration of haemodynamic variables. No significant increase in cTnI concentrations could be detected, but some cats showed values above the reference range.

## Figures and Tables

**Table 1:** Results of the pairwise comparison of heart rate and selected echocardiographic variables of six healthy male cats between baseline (BL), 20 min after intramuscular administration of 0.1 mg/kg medetomidine (MED) and 20 min after intramuscular administration of 0.5 mg/kg atipamezole (ATI) on day 0. The data are presented as median (range) and normally distributed variables are marked with *^n^*. Significantly different values from baseline are marked in bold and with * for *p* < 0.05, ** for *p* < 0.01 and *** for *p* < 0.001.

	Baseline	MED	ATI
Heart rate *^n^*	182 (150–230)	105 (81–143) ***	221 (127–260)
M-Mode- and 2D-derived variables			
Ejection fraction (%) *^n^*	66 (61–79)	29 (22–64) ***	62 (50–73)
End-diastolic volume (mL) *^n^*	4.7 (3–5.8)	6.3 (3.6–7.6) **	4.8 (3.5–6.9)
End-systolic volume (mL) *^n^*	1.6 (0.6–2.3)	3.6 (2.4–5.1) ***	1.9 (1.3–2.4)
Right ventricular fractional area change (%) *^n^*	61 (52–71)	46 (35–61) *	61 (51–67)
Left atrial fractional shortening (%) *^n^*	33 (29–38)	16 (11–19) ***	31 (22–38)
MAPSE (mm) *^n^*	5.5 (3.5–7.4)	2.9 (1.9–4.4) ***	4.2 (3.2–6)
LA:Ao *^n^*	1.3 (1.2–1–5)	1.4 (1.2–1.4)	1.4 (1.2–1.5)
Doppler-derived variables			
Stroke volume left ventricle (mL)	2.1 (1.4–2.9)	0.9 (0.7–1.9) **	1.7 (0.8–3.2)
IVRT (ms)	56 (49–60)	76 (54–88) **	48 (47–53)
Mitral valve E wave peak velocity (m/s)	0.62 (0.52–0.87)	0.45 (0.25–0.6) *	0.77 (0.62–1)
Tissue Doppler-derived variables			
MV s peak velocity (cm/s)^*n*^	6.6 (5.4–8.8)	4.2 (3.7–4.3) **	7.2 (6.3–10)
MV e peak velocity (cm/s) *^n^*	8.9 (6–16)	6 (3.1–7.7)	13 (9.6–15)

E wave: early diastolic left ventricular inflow wave; MAPSE: mitral annulus plane systolic excursion; MV e: early diastolic myocardial movement at the height of the mitral valve; MV s: systolic myocardial movement at the height of the mitral valve; 2D: two dimensional.

**Table 2:** Heart rate and echocardiographic variables of six healthy male cats at baseline (BL), 20 min after intramuscular administration of 0.1 mg/kg medetomidine (MED) and 20 min after intramuscular administration of 0.5 mg/kg atipamezole (ATI) on day 0. The data are presented as median (range).

	Baseline	MED	ATI
Heart rate	182 (150–230)	105 (81–143)	221 (127–260)
M-Mode- and 2D-derived variables			
Interventricular septal thickness in diastole (mm)	4 (3–4)	4 (3–4)	4 (3–5)
Left ventricular internal diameter in diastole (mm)	19 (12–20)	19 (14–19)	16 (14–20)
Left ventricular posterior wall thickness in diastole (mm)	4 (3–4)	4 (3–5)	4 (4–5)
Interventricular septal thickness in systole (mm)	6.5 (5–8)	6 (3–6)	7 (5–8)
Left ventricular internal diameter in systole (mm)	9.5 (6–13)	14 (11–17)	9.5 (7–12)
Left ventricular posterior wall thickness in systole (mm)	7.5 (6–8)	6 (4–7)	7.5 (6–8)
Fractional shortening (%)	46 (34–50)	26 (13–29)	40 (32–54)
Ejection fraction (%)	66 (61–79)	29 (22–64)	62 (50–73)
End-diastolic volume (mL)	4.7 (3–5.8)	6.3 (3.6–7.6)	4.8 (3.5–6.9)
End-systolic volume (mL)	1.6 (0.6–2.3)	3.6 (2.4–5.1)	1.9 (1.3–2.4)
Right ventricular internal diameter in diastole (mm)	9 (6–11)	9 (7–11)	8.5 (8–12)
Right ventricular area in diastole (cm^2^)	1.9 (1.4–2.3)	1.4 (1–2.2)	1.6 (1.2–2.3)
Right ventricular area in systole (cm^2^)	0.69 (0.44–1.1)	0.69 (0.45–1.3)	0.57 (0.45–1.2)
Right ventricular fractional area change (%)	61 (52–71)	46 (35–61)	61 (51–67)
Left atrial maximal diameter (mm)	15 (13–16)	17 (13–21)	15 (13–17)
Left atrial minimal diameter (mm)	9.8 (8–11)	14 (11–17)	10 (9.4–11)
Left atrial fractional shortening (%)	33 (29–38)	16 (11–19)	31 (22–38)
Right atrial maximal diameter (mm)	12 (10–16)	13 (12–14)	13 (12–13)
Doppler-derived variables			
Aortic velocity (m/s)	0.9 (0.7–1.1)	0.45 (0.35–0.73)	0.91 (0.48–1.4)
Aortic velocity time integral (cm^3^)	9.2 (7–10)	4.6 (3.5–8.6)	7.2 (4–13)
Aorta diameter (mm)	8.7 (7.9–9.6)	8 (8–9)	8.4 (7.4–9.8)
Aortic area (cm^2^)	2.4 (2–2.9)	2 (2–2.5)	2.2 (1.7–3)
Stroke volume left ventricle (mL)	2.1 (1.4–2.9)	0.9 (0.7–1.9)	1.7 (0.8–3.2)
Cardiac output left ventricle (L/minute)	0.4 (0.2–0.5)	0.1 (0.1–0.2)	0.4 (0.2–0.4)
Left ventricular ejection time (s)	143 (113–151)	157 (132–178)	122 (115–142)
Pulmonic valve velocity time integral (cm^3^)	6.8 (4.9–9.6)	3.5 (2.8–4.8)	5.8 (5.1–8.2)
Pulmonic valve diameter (mm)	9.1 (9–10)	9 (8.1–9.5)	8.8 (8–9.7)
Pulmonic valve area (cm^2^)	2.6 (2.5–3.1)	2.5 (2.1–2.8)	2.4 (2–3)
Stroke volume right ventricle (mL)	1.8 (1.2–3)	0.85 (0.6–1.4)	1.5 (1–1.9)
Cardiac output right ventricle (mL/minute)	0.3 (0.3–0.6)	0.1	0.3 (0.2–0.5)
Mitral valve E wave peak velocity (m/s)	0.62 (0.52–0.87)	0.45 (0.25–0.6)	0.77 (0.62–1)
Mitral valve velocity time integral (cm^3^)	5.5 (3.6–7)	4.5 (3–6.7)	5.3 (4.1–8)
E wave deceleration time (ms)	73 (55–86)	75 (55–88)	65 (50–72)
Isovolumic relaxation time (ms)	56 (49–60)	76 (54–88)	48 (47–53)
Left atrial area (cm^2^)	3.3 (2.7–3.9)	3.6 (2.7–4.5)	3.2 (2–4.2)
LA:Ao	1.3 (1.2–1–5)	1.4 (1.2–1.4)	1.4 (1.2–1.5)
MAPSE (mm)	5.5 (3.5–7.4)	2.9 (1.9–4.4)	4.2 (3.2–6)
TAPSE (mm)	9 (7–10)	6.5 (5–9)	7 (6–10)
Tissue Doppler-derived variables			
MV s peak velocity (cm/s)	6.6 (5.4–8.8)	4.2 (3.7–4.3)	7.2 (6.3–10)
MV e peak velocity (cm/s)	8.9 (6–16)	6 (3.1–7.7)	13 (9.6–15)
TV s peak velocity (cm/s) (n = 5)	11 (9.7–17)	6.3 (5.6–8.4)	14 (11–17)
TV e peak velocity (cm/s) (n = 5)	14 (11–23)	9.9 (7.0–10)	24 (11–29)
E:e	6.8 (5.4–9.5)	7.9 (6.5–8.4)	6.4 (4.4–7.3)

E wave: early diastolic left ventricular inflow wave; MAPSE: mitral annulus plane systolic excursion; MV e: early diastolic myocardial movement at the height of the mitral valve; MV s: systolic myocardial movement at the height of the mitral valve; TV e: early diastolic myocardial movement at the height of the tricuspid valve; TV s: systolic myocardial movement at the height of the tricuspid valve; 2D: two dimensional.

**Table 3:** Median (range) of the heart rate on day 0–28 of six healthy male cats undergoing repeated intramuscular sedation on day 0, 7, 14, 21 and 28. Measurements were performed at baseline (BL), 20 min after intramuscular administration of 0.1 mg/kg medetomidine (MED) and 20 min after intramuscular administration 0.5 mg/kg atipamezole (ATI). n.a., not available (n < 5), * n = 5.

	Day 0	Day 7	Day 14	Day 21	Day 28
BL	182 (150–230)	204 (162–240) *	197 (186–268)	204 (180–262)	220 (160–280)
MED	105 (81–143)	96 (77–136)	103 (80–160)	99 (81–125)	101 (74–129)
ATI	221 (127–260)	182 (118–264)	202 (172–246)	186 (132–240)	n.a.

**Table 4:** The occurrence and type of arrhythmias on day 0–28 of six healthy male cats undergoing repeated intramuscular sedation on day 0, 7, 14, 21 and 28. Heart rhythm was analysed at baseline (BL), 20 min after intramuscular administration of 0.1 mg/kg medetomidine (MED) and 20 min after intramuscular administration of 0.5 mg/kg atipamezole (ATI). On day 7–28, no ECG was performed at BL (n.a.).

cat		Day 0	Day 7	Day 14	Day 21	Day 28
1 ^#^	BL	VPC	n.a.	n.a.	n.a.	n.a.
	MED	-	-	-	-	-
	ATI	VPC	-	-	-	-
2 *	BL	-	n.a.	n.a.	n.a.	n.a.
	MED	-	-	VPC	-	-
	ATI	-	VPC	-	-	-
3 ^#^	BL	-	n.a.	n.a.	n.a.	n.a.
	MED	VPC, IVR	IVR	VPC, IVR	IVR	IVR
	ATI	-	SA	VPC	IVR	-
4 **	BL	-	n.a.	n.a.	n.a.	n.a.
	MED	-	-	VPC	-	SA
	ATI	-	-	-	-	-
5 ^# §^	BL	-	n.a.	n.a.	n.a.	n.a.
	MED	VPC	-	-	-	VPC, SA
	ATI	-	-	-	-	-
6	BL	-	n.a.	n.a.	n.a.	n.a.
	MED	IVR	IVR	-	IVR	SA
	ATI	-	-	-	-	-

SA: sinus arrhythmia; IVR: idioventricular rhythm; VPC: ventricular premature complex; -: no arrhythmia recorded; * cTnI concentration above reference range on day 0; ** cTnI concentration above reference range on day 0 and 28; ^#^ aortic valve insufficiency on day 0; ^§^ mitral valve insufficiency on day 0.

## Data Availability

Data presented in this study are available as Supplementary Materials.

## Supplementary Materials

The following supporting information can be downloaded at: https://www.mdpi.com/article/10.3390/ani13121909/s1, Table S1: Sedation Day 0–28, Table S2: Echocardiography Day 0.
